# Regulation of gene expression by FSP27 in white and brown adipose tissue

**DOI:** 10.1186/1471-2164-11-446

**Published:** 2010-07-22

**Authors:** De Li, Yinxin Zhang, Li Xu, Linkang Zhou, Yue Wang, Bofu Xue, Zilong Wen, Peng Li, Jianli Sang

**Affiliations:** 1 College of Life Sciences, Beijing Normal University Xinjiekouwai Street 19, Xichen District Beijing 100875, China; 2 Protein Science Laboratory of the Ministry of Education, School of Life Sciences, Tsinghua University, Qinghuayuan, Haidian District, Beijing 100084, China; 3 College of Life Science and Bioengineering, Beijing Jiaotong University, Shangyuancun 3, Haidian District, Beijing 100044, China; 4 Department of Biology, Hong Kong University of Science and Technology, Clearwater Bay, Kowloon, Hong Kong; 5 Department of Biochemistry, Hong Kong University of Science and Technology, Clearwater Bay, Kowloon, Hong Kong

## Abstract

**Background:**

Brown and white adipose tissues (BAT and WAT) play critical roles in controlling energy homeostasis and in the development of obesity and diabetes. The mouse Fat-Specific protein 27 (FSP27), a member of the cell death-inducing DFF45-like effector (CIDE) family, is expressed in both BAT and WAT and is associated with lipid droplets. Over-expression of FSP27 promotes lipid storage, whereas *FSP27 *deficient mice have improved insulin sensitivity and are resistant to diet-induced obesity. In addition, *FSP27*-deficient white adipocytes have reduced lipid storage, smaller lipid droplets, increased mitochondrial activity and a higher expression of several BAT-selective genes. To elucidate the molecular mechanism by which FSP27 controls lipid storage and gene expression in WAT and BAT, we systematically analyzed the gene expression profile of *FSP27-*deficient WAT by microarray analysis and compared the expression levels of a specific set of genes in WAT and BAT by semi-quantitative real-time PCR analysis.

**Results:**

BAT-selective genes were significantly up-regulated, whereas WAT-selective genes were down-regulated in the WAT of *FSP27-*deficient mice. The expression of the BAT-selective genes was also dramatically up-regulated in the WAT of *leptin/FSP27 *double deficient mice. In addition, the expression levels of genes involved in multiple metabolic pathways, including oxidative phosphorylation, the TCA cycle, fatty acid synthesis and fatty acid oxidation, were increased in the *FSP27-*deficient WAT. In contrast, the expression levels for genes involved in extracellular matrix remodeling, the classic complement pathway and TGF-β signaling were down-regulated in the *FSP27-*deficient WAT. Most importantly, the expression levels of regulatory factors that determine BAT identity, such as CEBPα/β, PRDM16 and major components of the cAMP pathway, were markedly up-regulated in the WAT of *FSP27-*deficient mice. The expression levels of these regulatory factors were also up-regulated in *leptin/FSP27 *double deficient mice. Interestingly, distinct gene expression profiles were observed in the BAT of *FSP27-*deficient mice. Taken together, these data suggest that the WAT of *FSP27-*deficient mice have a gene expression profile similar to that of BAT.

**Conclusions:**

FSP27 acts as a molecular determinant that controls gene expression for a diversity of metabolic and signaling pathways and, in particular, the expression of regulatory factors, including CEBPα/β, PRDM16 and components of the cAMP signaling pathway, that control the identity of WAT and BAT.

## Background

Adipose tissues play crucial roles in the development of obesity, with white adipose tissue (WAT) functioning as an energy storage organ and brown adipose tissue (BAT) functioning as an energy consumption organ [1]. BAT is especially abundant throughout the life-span of rodents and in hibernating mammals. In humans it was believed to present only in newborns and disappears in adults [2,3]. Interestingly, BAT and its increased activities in response to low temperature (16°C) can be detected in adult humans by ^18^F-fluorodeoxyglucose (^18^F-FDG) positron-emission tomography-computed tomography (PET-CT), and the response negatively correlates with their increasing body mass index [4-6]. Although WAT and BAT both express a set of genes that are involved in the regulation of lipolysis, fatty acid metabolism, triacylglycerol (TAG) storage and insulin sensitivity [7,8], BAT contains a large number of mitochondria and is functionally more important as a thermogenic tissue [9]. Uncoupling protein 1 (UCP1), which is uniquely expressed in BAT, plays an important role in the uncoupling of oxidative phosphorylation and the conversion of energy into heat to maintain normal body temperature. In addition to UCP1, type 2 iodothyronine deiondinase (DIO2) [10], elongation of very long chain fatty acid-3 (ELOVL3) [11-13], COX8b [14,15] and lipid storage droplet protein 5 (LSDP5) [16-18] are also expressed at high levels in BAT but low levels in WAT. PGC-1α [19,20], a co-activator that coordinates multiple physiological cues for mitochondrial biogenesis and activity, is highly expressed in BAT but only expressed at low levels in WAT [21]. TR3, a member of the nuclear receptor super-family, is expressed in brown adipocytes upon cold exposure and inhibits adipocyte differentiation [22,23]. In contrast, mesoderm-specific transcript (MEST) gene expression is markedly enhanced in the WAT of mice with diet-induced and genetically caused obesity [24]. Its expression levels are positively correlated with white fat mass, which may be related to its putative lipase activity [25]. Resistin-like molecule alpha (RETNLα), which shares homology with Resistin [26,27], has also been shown to be exclusively expressed in the stromal vascular fraction of WAT [28].

The exact origin of brown and white adipocytes and their developmental relationship is not clear. Brown and white adipocytes were previously considered to be derived from a common preadipocyte pool [29]. However, recent studies have suggested instead that brown adipocytes and muscle cells share a similar precursor, based on the observation that knock-down of *PRDM16*, a BAT-specific gene [15,30], switched the fate of brown adipocytes to that of muscle cells [31]. PRDM16 appears to be a co-factor for CEBPβ and PGC1α/β, which up-regulates the expression of many BAT-selective genes while down-regulating the expression of WAT-selective genes [15,32]. Differentiated WAT is able to develop a BAT-like phenotype under special conditions including cold exposure or after the administration of a β3-agonist to WAT depots [33-35]. PPARγ has been shown to be required for the conversion of WAT to a BAT-like tissue based on the observation that this conversion is blocked in cold-acclimatized PPARγ-mutant (P465L) mice [36]. Over-expression of the *CEBPβ *gene in *CEBPα*-deficient mice results in increased expression of the Gsα subunit, enhanced mitochondrial biogenesis and increased UCP1 expression in WAT [37]. The WAT in mice over-expressing FOXC2 is converted into a BAT-like tissue with a corresponding increase in the expression levels of PGC1, UCP1 and cAMP pathway proteins [38]. Although many factors and regulatory pathways have been shown to play important roles in regulating the conversion of WAT to a tissue with a BAT-like phenotype and function, the upstream signals or factors that determine the fate of BAT vs. WAT and initiate the conversion of WAT to a BAT-like tissue remain unclear.

CIDE proteins, including CIDEA, CIDEB and Fat specific protein 27 (FSP27, also known as CIDEC in humans), have been identified as important regulators of various metabolic pathways [39]. Our previous work demonstrated that CIDEA is expressed at high levels in BAT, whereas CIDEB is expressed at high levels in liver. Mice with a deficiency in both CIDEA and CIDEB have a higher energy expenditure, enhanced insulin sensitivity and a resistance against high-fat-diet-induced obesity and diabetes [40,41]. The FSP27 protein was detected at high levels in WAT and at moderate levels in BAT [42]. Furthermore, CIDE family proteins have been shown to be associated with the lipid droplet enriched fraction [42-45] and over-expression of FSP27 promotes TAG storage [44]. *FSP27-*null mice have a lean phenotype and are resistant to diet-induced obesity [42,46]. In addition, the WAT of *FSP27*-deficient mice has smaller lipid droplets as well as an increased mitochondrial size and activity [42,46]. In particular, several genes that are preferentially expressed in the BAT (UCP1, DIO2 and CIDEA) and several regulatory factors (PPARα/γ, PGC1) are up-regulated in the WAT of *FSP27*-deficient mice [42]. Therefore, the WAT of *FSP27 *deficient mice appear to adapt certain features similar to those found in BAT. The regulatory pathways and the underlying mechanism of FSP27-mediated transcriptional regulation in WAT and BAT, however, remain unclear. Using microarray and semi-quantitative real-time PCR (qPCR) analyses, we demonstrated that the WAT of *FSP27 *or *FSP27/leptin *deficient mice have markedly increased expression of many BAT-selective genes and decreased expression of WAT-selective genes. In addition, the expression levels of many genes involved in mitochondrial oxidative phosphorylation, lipolysis, fatty acid oxidation and the TCA cycle were up-regulated in the WAT of *FSP27-*deficient mice, which is consistent with their increased mitochondrial activity and whole-body metabolism. In contrast, genes involved in the classic complement pathway, extracellular matrix remodeling and the TGF-β signaling pathway were down-regulated in the WAT of *FSP27-*deficient mice. Most importantly, the expression of regulatory factors that activate the expression of BAT-selective genes was up-regulated in the WAT of *FSP27 *deficient mice, providing a molecular explanation for the increased expression of BAT-selective genes and the acquisition of BAT-like properties in the WAT of *FSP27*-deficient mice.

## Results

### Alteration of the gene expression program in *FSP27-*deficient mice as determined by microarray analysis

To determine the extent of the physiological changes required to convert WAT into a BAT-like tissue and to systematically analyze the transcriptional program of WAT in *FSP27-*deficient mice, whole gene microarray analysis was performed using Affymetrix oligonucleotide arrays separately hybridized with RNA from the WAT of wild-type and *FSP27-*deficient null mice. The expression of approximately 22,700 transcripts represented on the Affymetrix mouse genome 430 2.0A microarray chips was quantified in pooled WAT samples from five each of the wild-type and *FSP27-*deficient mice. Duplicate hybridizations were performed for each sample. The genes were filtered according to the criteria of a decrease ≥ 30% (2^-0.5^) or increase ≥ 1.41-fold (2^0.5^) in *FSP27-*deficient mice and consistency between the duplicate experiments. After collapsing the dataset from adjusted probes to symbols by GSEA, the expression levels of 2,870 of 5,923 genes were changed in the WAT of *FSP27*^-/- ^mice (48.5%). Among them, 1,037 genes (36.1%) were increased, whereas 1,833 genes (63.9%) were decreased. Notably, the 10 most highly elevated genes (Table 1) include COX8b (40-fold increase), ELOVL3 (12-fold increase), TR3 downstream gene 2 (NDG2, 8-fold increase), CIDEA (8-fold increase) and cytochrome c oxidase subunit VIIa polypeptide (COX7A1, 6-fold increase). Interestingly, COX8b (a pseudogene), ELOVL3, CIDEA and COX7A1 have been reported to be highly expressed in BAT but not WAT [14,15]. Pyruvate dehydrogenase kinase isozyme 4 (PDK4), an enzyme involved in pyruvate metabolism [47], and the lipid droplet binding protein LSDP5 were also significantly increased. Overall, the 10 most up-regulated genes in the WAT of *FSP27*-deficient mice comprised mostly genes selectively expressed in BAT. In contrast, the 10 most down-regulated genes involved in diverse pathways, such as proteinase 3 (PRTN3) [48] and retinitis pigmentosa GTPase regulator (RPGR) [49]. Another of the 10 most down-regulated proteins is MEST, a protein expressed at high levels in WAT but not in BAT [24].

**Table 1:** The ten most up-regulated and ten most down-regulated genes in the WAT of *FSP27*-null mice

Name	Gene Title	Fold
Ten most upregulated genes		
COX8B	cytochrome c oxidase, subunit 8B pseudogene	40.06
ELOVL3	elongation of very long chain fatty acids (FEN1/Elo2, SUR4/Elo3, yeast)-like 3	11.9
NDG2	Nur77 downstream gene 2	8.35
CIDEA	cell death-inducing DFFA-like effector a	8.22
COX7A1	cytochrome c oxidase subunit VIIa polypeptide 1 (muscle)	6.74
CA1	carbonic anhydrase I	6.72
PDK4	pyruvate dehydrogenase kinase, isozyme 4	5.83
CCRN4L	CCR4 carbon catabolite repression 4-like (*S. cerevisiae*)	5.69
NAP1L5	nucleosome assembly protein 1-like 5	5.63
9530008L14RIK	RIKEN cDNA 9530008L14 gene	5.59
Ten most downregulated genes		
PRTN3	proteinase 3 (serine proteinase, neutrophil, Wegener granulomatosis autoantigen)	-26.45
RPGR	retinitis pigmentosa GTPase regulator	-23.68
IGL-V1	immunoglobulin lambda chain, variable 1	-18.31
MEST	mesoderm specific transcript homolog (mouse)	-14.68
EAR11	eosinophil-associated, ribonuclease A family, member 11	-13.50
Tsr2	TSR2, 20S rRNA accumulation, homolog (*S. cerevisiae*)	-13.15
NAP1L3	nucleosome assembly protein 1-like 3	-12.85
MS4A6D	membrane-spanning 4-domains, subfamily A, member 6D	-11.98
SFRP5	secreted frizzled-related protein 5	-11.88
TOP2A	topoisomerase (DNA) II alpha 170 kDa	-10.89

**Figure 1:**
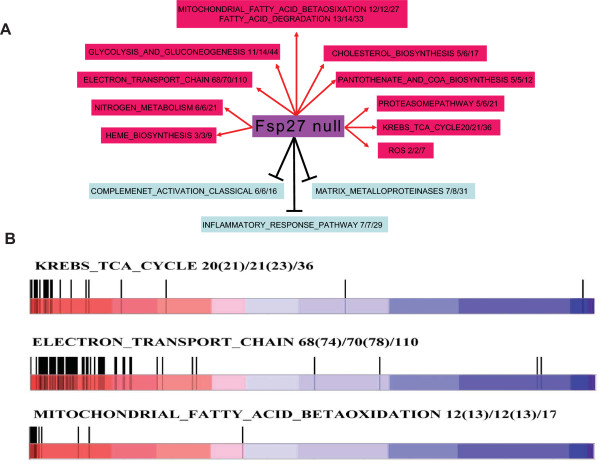
**Dramatically altered transcriptional network in the WAT from *FSP27-*deficient mice by microarray analysis**. **A) **The most significant up-regulated and down-regulated pathways in the WAT of *FSP27*-null mice were identified using Gene Set Enrichment Analysis (GSEA). The last number of each pathway represents the total number of genes in one gene set. The middle number represents the number of genes with an altered expression pattern (using the criteria described above), and the first number represents the number of up-regulated or down-regulated genes in the gene set. **B) **The pathways that were significantly up-regulated, as revealed by enrichment plot analysis, were the Krebs-TCA cycle, the electron transport chain and the mitochondrial fatty acid β oxidation pathway. The denotation of the numbers in this figure is as follows: The number of genes having a higher than 2^0.5^-fold increase (the number of genes having a higher than 1-fold increase in expression)/the number of genes with an altered expression pattern as determined by microarray in the WAT of *Fsp27*-/- mice (total number of genes detected by the microarray)/total number of genes in a specific pathway.

The pathways that are altered in the WAT of *FSP27-*deficient mice were determined by GSEA analysis. There were extensive changes in the expression of genes involved in many different metabolic pathways in the WAT of *FSP27-*deficient mice. Most strikingly, the expression of many proteins that localize to mitochondria were significantly up-regulated (Figures 1A &1B and Additional file 1). These genes are involved in the Krebs/TCA cycle (20 of 36 genes were up-regulated), the electron transport chain (ETC, 68 of 110 genes were up-regulated), mitochondrial fatty acid β-oxidation (12 of 17 genes were up-regulated) and fatty acid degradation (13 of 33 genes were up-regulated). These data are consistent with our previous observation that mitochondrial biogenesis and activity is increased in the WAT of *FSP27-*deficient mice [42]. In addition, the mRNA levels of many genes involved in glycolysis, gluconeogenesis, cholesterol biosynthesis, pantothenate and CoA biosynthesis, heme biosynthesis, nitrogen metabolism, ROS generation and the proteasome pathway were significantly up-regulated (Figure 1A, and Additional file 1). Microarray analysis also demonstrated that some of the proteins normally expressed in BAT, such as PGC1α, UCP1 and DIO2, were expressed at high levels in the WAT of *FSP27-*deficient mice. Furthermore, factors that inhibit BAT differentiation, such as RB/p107, were down-regulated in the WAT of *FSP27*^**-/- **^mice (Additional file 2). These data are consistent with our previous observations [42].

The classic complement pathway, which plays an important role in the initiation of the inflammatory response, was significantly down-regulated in the WAT of *FSP27*-deficient mice (6 of 16 genes were down-regulated, including complement component genes C1QA, C1QB, C1R, C1S, C2 and C6 (Additional file 2). Extracellular matrix proteins, including 15 members of the collagen family in particular, were significantly down-regulated (Additional file 3). Matrix metalloproteinase pathway (MMP) genes, including matrix metallopeptidases 7, 14 and 16 and the tissue inhibitor of metallopeptidase (TIMP) 1-4 (Additional file 3), were also significantly down-regulated. Some genes involved in the TGF-β pathway were also down-regulated (Additional file 3). Furthermore, microarray analysis also showed that IRS3, AKT2 and GLUT4, three genes that are crucial for the regulation of insulin sensitivity, were significantly up-regulated (Additional file 2). These data are in accordance with our previous observation that *FSP27-*deficient mice had increased insulin sensitivity [42].

**Figure 2:**
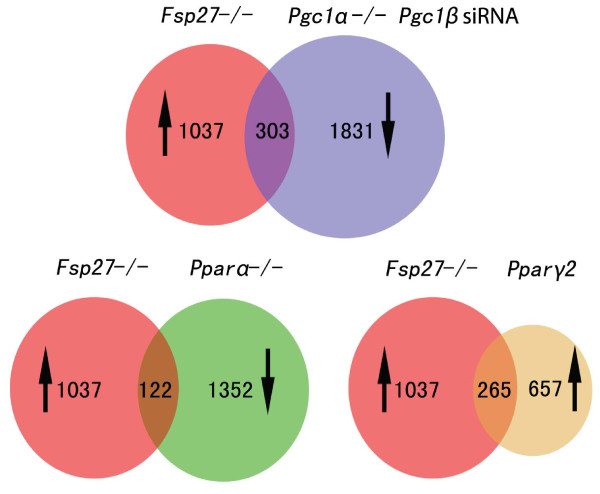
**Comparison of the gene expression profile of *FSP27-*deficient mouse white adipocytes with that of *Pgc1α/Pgc1β *double deficient mouse brown adipocytes, the *Pparα-*deficient mouse WAT or the cells over-expressing PPARγ2**. The up-regulated genes (↑) in the white adipocytes of *FSP27-*null mice were compared with the following: 1) the down-regulated genes (↓) in Pgc1α^-/- ^differentiated brown adipocytes expressing a siRNA specific for Pgc1β (Pgc1α^-/- ^PGC1βsiRNA), 2) the down-regulated genes (↓) in the WAT of *Pparα*^-/- ^mice (*Pparα*^-/-^) or 3) the up-regulated genes (↑) in the PPARγ2 over-expressing NIH-3T3 cells (PPARγ2). The number in the over-lapping area represents the number of genes that have conserved changes between the two cell types in the comparison.

The profound increase in mitochondrial activity and the up-regulation of mitochondrial proteins can likely be attributed to the increased expression of PGC1, PPARα and PPARγ, as had been previously seen in the WAT of *FSP27-*deficient mice. The gene expression profile in the WAT of *FSP27-*deficient mice was compared with that of differentiated brown fat cells that were PGC1α- and PGC1β-deficient using Gene Set Enrichment Analysis (GSEA) [50]. Among the 1,037 transcripts that were significantly up-regulated in the WAT of *FSP27-*deficient mice, a significant percentage of them (303, 29.2%) were down-regulated in the PGC1α^-/- ^differentiated brown fat cells expressing an siRNA specific for PGC1β knockdown, suggesting that those 303 genes are downstream targets of PGC1α and PGC1β (Figure 2). Comparison of the gene expression profile in the WAT of *FSP27-*deficient mice with that in PPARγ2 over-expressing NIH3T3 cells and the WAT of *PPARα*-deficient mice, 265 of the 1,037 (25.6%) genes were significantly up-regulated in the PPARγ2 over-expressing NIH3T3 L1 cells, whereas 122 of the 1,037 (11.8%) genes were down-regulated in the WAT of *PPARα *null mice (Figure 2). These data suggest that FSP27 acts upstream to control the expression of regulatory factors such as PGC1α and PPARα/γ and thus affect the expression of the downstream targets of these regulatory factors, such as UCP1, DIO2 and CIDEA.

### Validation of the gene expression program in the WAT and BAT of *FSP27*-deficient mice by quantitative real-time PCR analysis

**Figure 3:**
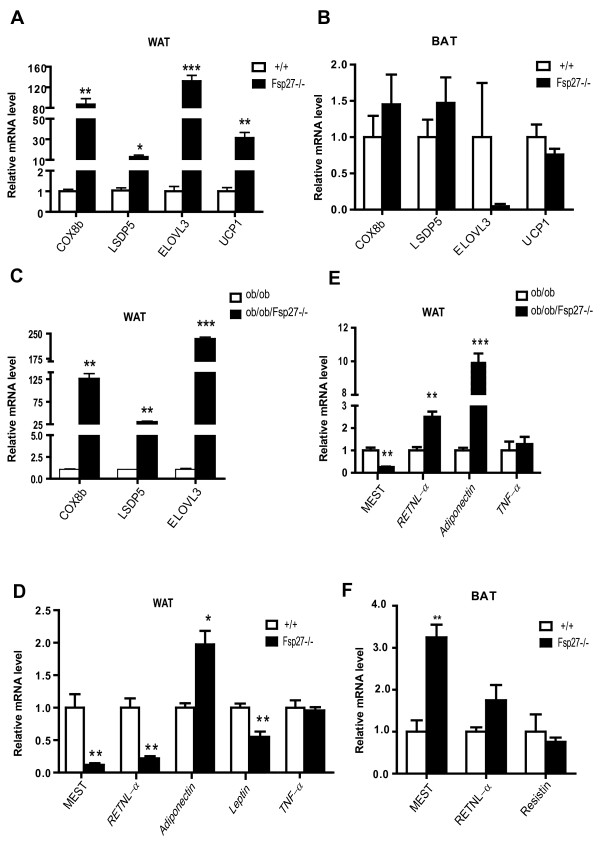
**Up-regulation of BAT-selective genes and down-regulation of WAT-selective genes in the WAT of *FSP27-*deficient mice**. (**A & B**) Relative expression levels of BAT-specific genes (COX8b, LSDP5, ELOVL3 and UCP1) in the WAT (A) and BAT (B) of wild-type (+/+) and *FSP27-*deficient (*FSP27*^-/-^) mice. (**C**) Expression levels of BAT-specific genes in the WAT of leptin-deficient (*ob/ob*) and *leptin/FSP27 *double-deficient (*ob/ob/FSP27*^-/-^) mice. (**D & E**) Relative mRNA levels of WAT-selective genes (MEST, RETNLα, leptin, adiponectin and TNFα ) in the WAT of wild-type (+/+) and *FSP27*-null (*FSP27*^-/-^) mice, leptin-deficient (*ob/ob*) and *leptin/FSP27 *double-deficient (*ob/ob/FSP27*^-/-^) mice (**D**) and in the BAT of wild-type (+/+) and *FSP27*-null (*FSP27*^-/-^) mice. (**E**). The bars represent the mean ± S.E.M. ***P < 0.001, **P < 0.01 and *P < 0.05. The expression levels of each gene were assessed by semi-quantitative real-time PCR analysis. Three-month-old male mice were used in the analyses depicted in this and following figures unless otherwise noted.

To validate our microarray data, qPCR analysis was used to analyze the expression levels of representative genes taken from a variety of pathways in the WAT and BAT of *FSP27- *deficient mice. In a consistent manner, the expression levels of genes selectively expressed in BAT, including COX8b, ELOVL3 and UCP1, were dramatically up-regulated in the WAT of *FSP27-*deficient mice (85-, 140- and 35-fold increases, respectively; Figure 3A). The expression levels of LSDP5 were also up-regulated (12-fold increase) in *FSP27-*deficient WAT. Given that *FSP27 *is also expressed in BAT, the expression levels of these genes were then examined in the BAT of *FSP27-*deficient mice. Surprisingly, there were similar levels of COX8b, LSDP5 and UCP1, but reduced expression of ELOVL3 in the BAT of *FSP27*^-/- ^mice (Figure 3B). Because the expression levels of many genes in various metabolic pathways are affected by obesity, we generated *leptin *and *FSP27 *double deficient mice *(ob/ob/FSP27*^-/-^) and examined the expression levels of BAT-selective genes in the WAT of these animals. Consistent with the previous observations, the levels of COX8b, LSDP5 and ELOVL3 were significantly elevated (125-, 30- and 230-fold increases) in the WAT of *ob/ob/FSP27 *deficient mice compared with that of *ob/ob *mice (Figure 3C), suggesting that the WAT of *ob/ob/FSP27*^-/- ^mice also acquired certain BAT-like properties despite the significantly higher lipid accumulation in these obese mice.

The expression levels of genes selectively expressed in the WAT were also examined by qPCR, which revealed that the mRNA levels of MEST were significantly reduced (approximately 10-fold lower compared with that of wild-type mice, Figure 3D) in the WAT of *FSP27-*deficient mice. qPCR on another WAT-selective gene, resistin-related protein alpha (RETNLα), revealed significantly lower mRNA levels for RETNLα (5-fold decrease) in the WAT of *FSP27-*deficient mice, suggesting that the expression of WAT-selective genes was suppressed in *FSP27*-deficient WAT. As an endocrine organ, WAT can secrete various adipocytokines and inflammatory cytokines, such as adiponectin, leptin and TNFα. There was an increase in adiponectin levels and a reduction in leptin expression in the WAT of *FSP27*-deficient mice, which is consistent with their lean and insulin sensitive phenotypes (Figure 3D). No difference in the expression of TNFα was observed between wild-type and *FSP27-*deficient WAT (Figure 3D). Down-regulation of MEST (4-fold decrease) and up-regulation of adiponectin (10-fold decrease) were also observed in the WAT of *ob/ob/FSP27*^-/- ^mice compared with *ob/ob *mice. Surprisingly, the mRNA levels of RETNLα were up-regulated in the WAT of *ob/ob*/*FSP27*^-/- ^mice (2.5-fold increase, Figure 3E). The expression levels of MEST and RETNLα in the BAT of *FSP27-*deficient mice were higher than those of wild-type mice (3.2- and 1.75-fold increases for MEST and RETNLα, respectively; Figure 3F). Previous reports showed that ectopic expression of MEST markedly enlarged the size of adipocytes and that its expression levels were positively correlated with larger adipocytes [24]. Increased MEST expression is consistent with our previous observation that the BAT of *FSP27*-deficient mice had larger lipid droplets and increased TAG accumulation [42]. The expression levels of resistin, another WAT-selective gene, were similar in the BAT of both wild-type and *FSP27-*deficient mice (Figure 3F).

**Figure 4:**
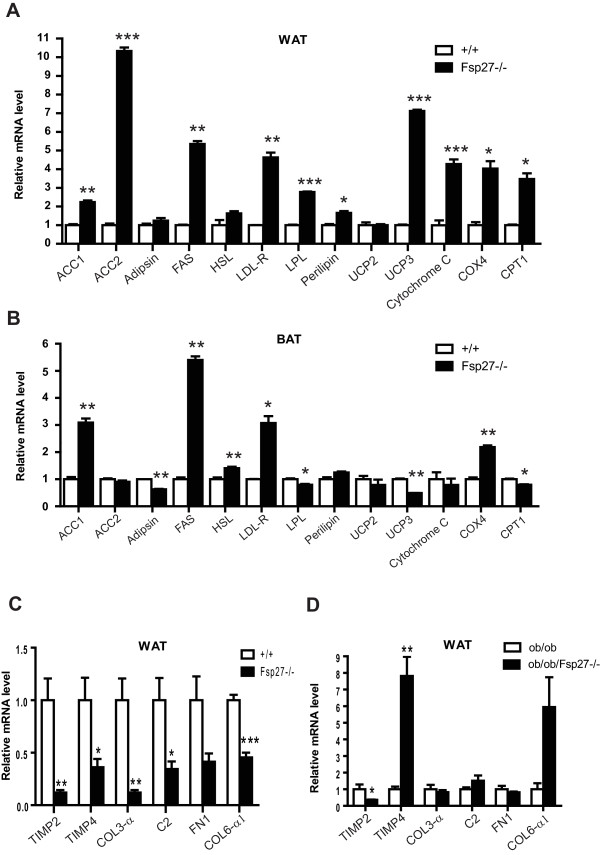
**Altered gene expressions in various metabolic pathways in the WAT of *FSP27-*deficient**. (**A & B**) Relative mRNA levels of genes in various metabolic pathways in the WAT (**A**) and BAT (**B**) of wild-type (+/+) and *FSP27-*deficient (*FSP27*^-/-^) mice. (**C & D**) Relative mRNA levels of genes in the classic complement and extracellular matrix remodeling pathways in the WAT of wild-type (+/+) and *FSP27*-null (*FSP27*^-/-^) mice (**C**) and of *leptin*-deficient (*ob/ob*) and *leptin/FSP27 *double-deficient (*ob/ob/FSP27*^-/-^) mice (**D**). The bars represent the mean ± S.E.M. ***P < 0.001, **P < 0.01 and *P < 0.05.

The expression levels of genes involved in various metabolic pathways, including lipid metabolism, uncoupling activity and mitochondrial electron transport chain activity, were then examined. The expression levels of genes involved in the fatty acid synthesis pathway, including ACC1 (2.5-fold increase), ACC2 (10-fold increase), and fatty acid synthase (FAS, 5-fold increase), were up-regulated in the WAT of *FSP27*^-/- ^mice (Figure 4A). The expression levels of genes involved in the mitochondrial oxidative pathway (Cyto-C, COX4 and CPT1) and the lipoprotein pathway, including the LDL receptor (LDLR, 4-fold increase) and Lipoprotein lipase (LPL, 2.6-fold increase), were significantly up-regulated in the WAT of *FSP27-*deficient mice (Figure 4A). Interestingly, UCP3, a mitochondrial uncoupling protein that is homologous to UCP1, is also significantly increased, suggesting an increase in the uncoupling activity of the WAT of *FSP27-*deficient mice. The expression levels of ACC1, FAS, HSL, LDLR and COX 4 were also up-regulated in the BAT of *FSP27*^-/- ^mice, whereas the mRNA levels for UCP3, CPT1, LPL and adipsin were down-regulated in the BAT of *FSP27-*deficient mice (Figure 4B).

To determine whether the expression levels of genes in the classic complement and extracellular matrix remodeling pathways were indeed reduced in the WAT of *FSP27*^-/- ^mice, as indicated by the microarray analysis, the expression levels of complement factor 2 (C2), TIMP2/4, Fibronectin1 (FN1), Collagen 3 alpha (COL3-α) and 6 alpha1 (COL6-α1) were measured by qPCR. The levels of TIMP2 and TIMP4 were significantly reduced in the WAT of *FSP27-*deficient mice (90% and 70% reduction for TIMP2 and TIMP4, respectively; Figure 4C). The levels of C2, COL3-α and COL6-α1 were also reduced in the *FSP27-*deficient WAT (70%, 90% and 50% reductions, respectively; Figure 4C). Interestingly, lower levels of TIMP2 (70% lower) but higher levels of TIMP4 (7.8-fold higher) and COL6-α1 (6 fold higher) were observed in the WAT of *ob/ob/FSP27-*deficient mice (Figure 4D). No differences in the levels of COL3α1, C2 or Fibronectin1 were observed between *ob/ob *and *ob/ob*/*FSP27*^-/- ^mice (Figure 4D).

**Figure 5:**
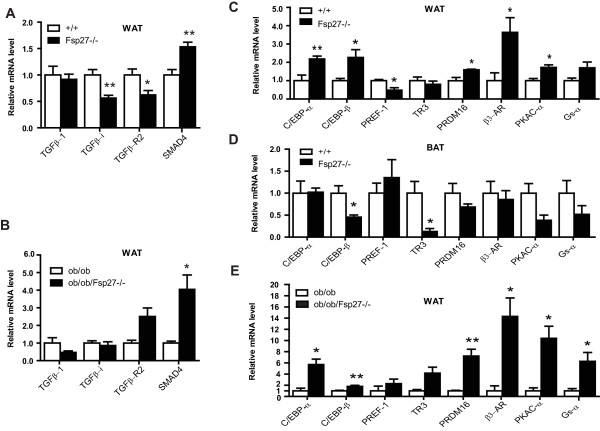
**Expression levels of regulatory genes in the WAT of *FSP27-*deficient mice**. (**A & B**) Relative mRNA levels of genes in the TGF-β pathway in the WAT of wild-type (+/+) and *FSP27*-null (*FSP27*^-/-^) mice (**A**) and leptin-deficient (*ob/ob*) and *leptin/FSP27 *double-deficient (*ob/ob/FSP27*^-/-^) mice (**B**). (**C & D & E**) Relative mRNA levels of regulatory factors in the WAT (**C**) and the BAT (**D**) of wild-type (+/+) and *FSP27*-null (*FSP27*^-/-^) mice and in the WAT of leptin-deficient (*ob/ob*) and *leptin/FSP27 *double-deficient (*ob/ob/FSP27*^-/-^) mice (**E**). The bars represent the mean ± S.E.M. **P < 0.01 and *P < 0.05.

Given that lipid metabolism and mitochondrial activity are controlled by many regulatory factors in WAT and BAT, the expression levels of genes involved in the TGF-β and cAMP pathways and of genes involved in the regulation of adipogenesis were analyzed. While the mRNA level of TGF-β1 was similar in the WAT of both wild-type and *FSP27-*deficient mice, the mRNA levels for TGF-β-receptor 2 (TGFβ-R2) and TGFβ-induced protein (TGF-βi), an extracellular matrix molecule induced by TGF-β that mediates the adhesion and spreading of different cell types [51], were significantly down-regulated in the WAT of *FSP27-*deficient mice (40% and 50% reduction, respectively; Figure 5A). Interestingly, SMAD4, a downstream mediator of the TGF-β pathway, was significantly up-regulated (Figure 5A) in the WAT of *FSP27-*deficient mice. The expression levels of SMAD4 were also increased in the WAT of *ob/ob/FSP27*^-/- ^mice compared with that of *ob/ob *mice (Figure 5B), whereas no difference in the expression levels of TGFβ-R2 and TGF-βi was observed in the WAT of *ob/ob *and *ob/ob/FSP27*^-/- ^mice (Figure 5B).

**Figure 6:**
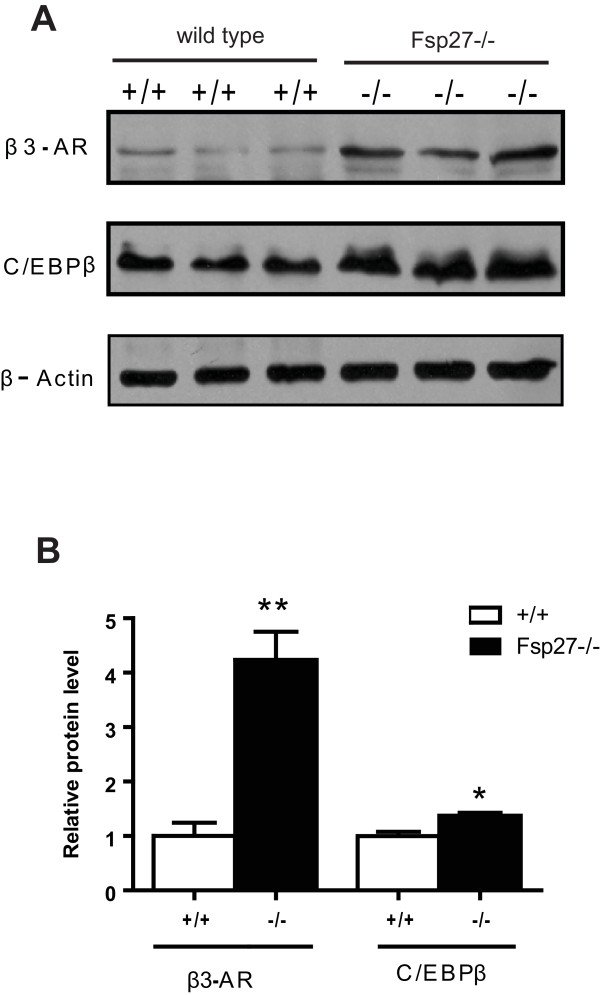
**Increased C/EBPβ and μ3-AR protein levels in the WAT of *FSP2*7-null mice**. **(A) **Western blot showing the increased protein levels of C/EBPβ and β3-AR in the WAT of *FSP27-*deficient mice (-/-) compared with those of wild type mice (+/+). β-actin was used as a loading control. (B) The intensity of the indicated protein bands in **(A) **was quantified using Quantity One software (BioRad, USA) and used for statistical analyses. The western blot analyses were performed in triplicate. The relative protein levels in the wild type mice were designated as 1.0. **P < 0.01 and *P < 0.05.

The expression levels of several regulatory factors, including CEBPα and CEBPβ, PREF-1, PRDM16, TR3 and components of the cAMP pathway, were further analyzed. The expression levels of CEBPα and CEBPβ, which are transcriptional factors controlling adipocyte differentiation and PPARγ expression, were significantly increased in the WAT of *FSP27-*deficient mice (Figure 5C), which is consistent with the increased PPARγ expression [42]. In contrast, the expression levels of CEBPβ were significantly reduced in the BAT of *FSP27-*deficient mice (Figure 5D). Interestingly, the expression of PREF-1, a unique preadipocyte marker, was reduced in the WAT of *FSP27-*deficient mice, whereas its expression level in the BAT was similar between wild-type and *FSP27*^-/- ^mutant mice (Figure 5C &5D). The expression levels of PRDM16 in the WAT of *FSP27*^-/- ^mice were also significantly up-regulated compared with that of wild-type mice. The expression of TR3 was decreased in the BAT of *FSP27*^-/- ^mice (Figure 5D). Importantly, there was a significantly increased expression of the β3-adrenergic receptor (β3-AR, 3.5-fold increase, Figure 5C), the protein kinase A catalytic subunit-α (PKAC-α, 1.8-fold increase) and the Gs alpha subunit (Gs-α). The increased expression of genes involved in the cAMP pathway could contribute to the activated metabolism and increased UCP1 expression [42] found in the WAT of *FSP27*-deficient mice. The expression levels of CEBPα/β, PRDM16, β3-AR, PKAC-α and Gs-α were also up-regulated in the WAT of *ob/ob/FSP27*^-/- ^mice (Figure 5E), which is consistent with the increased expression of BAT-selective genes in these mice. Furthermore, western blot analysis indicated that the protein levels of CEBPβ and β3-AR were significantly increased (1.5- and 4-fold increases for CEBPβ and β3-AR, respectively; Figure 6), which is consistent with their increased mRNA levels. The increased expression of BAT-selective genes (CIDEA and COX8b), CEBPβ and β3-AR was also observed in the WAT of young female and old male *FSP27-*deficient mice (Additional file 4), suggesting that the acquisition of BAT-like properties in the WAT of *FSP27-*deficient mice occurs regardless of sex or age. Interestingly, the increased expression of PRDM16 was observed only in the WAT of young male and female *FSP27*^-/- ^mice but not in the WAT of old mice, suggesting that the regulation of PRDM16 expression is age-dependent.

## Discussion

Using microarray and qPCR analyses, we demonstrated that FSP27 plays an important role in regulating mitochondrial oxidative phosphorylation, adipocyte differentiation, lipolysis, fatty acid oxidation, the inflammatory response and the extracellular matrix structure by controlling extensive gene expression programs in both WAT and BAT. Semi-quantitative real-time PCR analyses validated the reliability of the microarray data (Additional file 5). Importantly, genes that are highly enriched in BAT (e.g., COX8b, ELOVL3 and LSDP5) were drastically up-regulated in the WAT of *FSP27-*deficient mice. In contrast, WAT-enriched genes (e.g., MEST and RETNLα) were significantly down-regulated. The expression levels of the set of WAT-selective genes that were defined by Kajimura et al [30] were specifically examined in our microarray analyses (Additional file 6). A subset of these genes is down-regulated but others are up-regulated, suggesting that these genes are controlled by different mechanisms. The BAT-like phenotype of *FSP27-*deficient WAT was further supported by its significantly elevated expression of many genes involved in the regulation of the TCA cycle, the electron transport chain, uncoupling activity and the fatty acid oxidation pathway, resulting in its conversion from an energy storage organ to an energy consumption organ. The increased expression of BAT-selective genes and enhanced expression of genes involved in various metabolic pathways in the WAT of *FSP27*^-/- ^mice are likely due to the up-regulation of several regulatory factors: 1) CEBPα and CEBPβ, which activate PPARγ expression and promote adipogenesis; 2) PRDM16, which promotes the differentiation of preadipocytes and myoblasts into brown adipocytes; 3) PPARα/γ and PGC1 and their downstream target genes [42]; and 4) several genes in the cAMP signaling pathway (e.g., β3-AR, PKAC-α and Gs-α), which promote the conversion of white to brown adipocytes by inducing UCP1 expression and mitochondrial activity in white adipose depots. Therefore, the up-regulation of CEBPα/β and PRDM16 may act as an initial step to increase the expression of PPARα/γ and PGC1 and promote adipocyte differentiation. The increased expression of PPARα/γ, PGC1 and the proteins involved in cAMP signaling in conjunction with the reduced expression of Rb, P107 and RIP140 [42] may act in concert to up-regulate genes specifically expressed in BAT and genes involved in energy metabolism, which in turn would promote the conversion of WAT to a BAT-like tissue in *FSP27-*deficient mice.

The underlying mechanism responsible for the increased expression of CEBPβ and PRDM16 in the WAT of *FSP27*-deficient mice is not understood. PREF-1, which inhibits adipocyte differentiation via the upregulation of SOX9, was down-regulated, likely resulting in a reduction in the expression levels of SOX9 [52,53]. SOX9 can bind to the promoters for CEBPβ and CEBPδ, which would consequently suppress the activity of those promoters resulting in the reduced expression of PREF-1 and possibly SOX9 in the WAT of *FSP27- *deficient mice. This cascade of events could, therefore, lead to the up-regulation of CEBPβ/δ. In addition, adiponectin was up-regulated in the WAT of *FSP27*-deficient mice (Fig. 3D). Adiponectin and its receptors have been reported to induce extracellular Ca^2+ ^influx and activate Ca^2+ ^/calmodulin-dependent protein kinase kinase beta (CaMKKβ), AMPK and SIRT1, resulting in an increase in the expression of PGC-1α [54]. Therefore, the up-regulation of adiponectin may contribute to the increased levels of BAT-specific genes. In addition, CEBPβ has been shown to be phosphorylated and activated in response to an increase in the intracellular calcium concentration, which was caused by the activation of CaMKKβ [55]. The mechanism responsible for the transcriptional regulation of CEBPβ and PRDM16 in the WAT of *FSP27 *deficient mice remains to be clarified.

Our previous study indicated that the conversion of WAT to a BAT-like tissue in *FSP27-*deficient mice could be partially recapitulated in differentiated mouse embryonic fibroblasts (MEFs) isolated from *FSP27*-deficient mice. The differentiated *FSP27-*deficient MEFs showed characteristics such as increased lipolysis, smaller but multiple lipid droplets and reduced TAG storage. More importantly, the *FSP27-*deficient MEFs had an increased rate of fatty acid oxidation and higher expression levels of PGC1α, CIDEA, UCP1 and COX4 in the presence of T3 [42]. The relative expression levels of these BAT-selective genes in differentiated *FSP27-*deficient MEFs, however, were not as high as those seen in the WAT of *FSP27-*deficient mice [42]. For example, the mRNA levels of CIDEA and UCP1 are similar between wild-type and *FSP27*^-/- ^MEFs, and the level of COX4 was 1.5-fold higher in *FSP27*^-/- ^MEFs compared with that of wild-type cells. In the *FSP27-*deficient WAT, in contrast, mRNA levels of these three genes were 2.6-, 16.8- and 2.3-fold increased, respectively [42]. In addition, there was no difference in the expression of BAT-selective genes and mitochondrial activity between differentiated wild-type and FSP27 knock-down 3T3-L1 cells (data not shown). In differentiated FSP27 knock-down 3T3-L1 cells, Keller et al. also observed no difference in the expression of BAT-selective genes [56]. The discrepancy between the WAT of *FSP27-*deficient mice and *in vitro *cultured *FSP27 *deficient adipocytes may be due to the lack of crucial extracellular factors that cooperate with FSP27 to determine the BAT identity in cultured adipocytes. Alternatively, the commitment to the transition of WAT into BAT-like tissue in *FSP27*^-/- ^mice may occur before differentiation at the precursor stage. Further experiments will be needed to distinguish these possibilities.

Interestingly, there was a significantly reduced expression of genes involved in TGF-β signaling in the WAT of *FSP27*^-/- ^mice. Because activation of the TGF-β signaling pathway was shown to inhibit adipocyte differentiation [57], reduced TGF-β signaling may further enhance white adipocyte differentiation in *FSP27-*deficient mice. The classic complement pathway, which plays a key role in the initiation of the inflammatory response in adipose tissue under obese and insulin-resistant conditions [58], was significantly down-regulated in the WAT of *FSP27-*deficient mice, implicating a reduced inflammatory response in the WAT. These data were also consistent with our previous observation that *FSP27*-deficient mice had improved insulin sensitivity and a lean phenotype [42,46]. Finally, a significantly reduced expression of collagen family proteins, MMPs and TIMPs, which all play key roles in determining the three-dimensional (3-D) structure of the WAT and in controlling extracellular matrix (ECM) remodeling [59-62], was observed in the WAT of *FSP27-*deficient mice. These data suggest that the 3-D structure and, in particular, the ECM structure of *FSP27-*deficient WAT is different from that of wild-type mice, which may be reflected in its reduced adipocyte size and reduced inflammatory response. As main components of extracellular matrix, the levels of collagen family proteins are generally up-regulated in the adipose tissue of diabetic mice [60]. In addition, animals with a disruption of collagen VI, a predominant collagen in adipose tissue, have larger adipocytes but improved insulin sensitivity [62]. The decreased ECM pathway may contribute to the reduced lipid storage in white adipocytes and the improved insulin sensitivity in *FSP27-*deficient mice.

Using *leptin/FSP27 *double deficient mice as a model system, the expression of BAT-selective genes and regulatory factors was analyzed under the conditions of *FSP27 *deficiency and obesity. BAT-selective genes and key metabolic regulators (e.g., CEBPα/β and PRDM16) and members of the cAMP signaling pathway (e.g., β3-AR, PKAC-α and Gs-α) were all up-regulated in the *FSP27/leptin *double deficient mice, which is consistent with that seen under the condition of *FSP27 *deficiency alone. Thus, white adipocytes in *FSP27 and leptin *double deficient mice also acquire BAT-like properties and become an energy consuming organ. The expression profile of the genes involved in TGF-β signaling, extracellular matrix remodeling and the classic complement pathway in the WAT of *ob/ob/FSP27*^-/- ^mice, however, was different from that of *FSP27 *deficient mice. This observation indicated that gene expression in these pathways in obese animals requires the cooperative action of FSP27 and other extrinsic factors.

Paradoxically, the gene expression profile in the BAT of *FSP27-*deficient mice was dramatically different from that of the *FSP27-*deficient WAT based on the following observations: 1) in the BAT of *FSP27-*deficient mice, there was a significantly increased expression of WAT-selective markers (MEST and RETNLα) that are normally suppressed by the expression of PRDM16; 2) the expression of several mitochondrial genes (UCP3 and CPT1) was down-regulated; and 3) the expression levels of regulatory factors including CEBPβ and TR3 were reduced in the BAT of *FSP27-*deficient mice, whereas the expression of components in the cAMP pathway was similar to that of wild-type mice. The mechanism by which the expression profile of BAT of *FSP27-*deficient mice differs from that of WAT remains unclear. Given that CIDEA is expressed at a high level in BAT, it may replace FSP27 and perform some of the functions of FSP27. Further analysis using *CIDEA/FSP27 *double knock-out mice will be needed to address the role of these individual genes in BAT.

## Conclusions

Overall, our data suggest that FSP27 acts as a crucial factor that controls the expression of genes involved in various regulatory and metabolic pathways in WAT and BAT. FSP27 deficiency results in the up-regulation of regulatory factors that promote the activation of BAT-selective genes and genes involved in energy expenditure processes such as mitochondrial activity. The coordinated regulation of such profound transcriptional networks that affect multiple metabolic and signaling pathways is an interesting property of *FSP27*-deficient mice. Although the precise mechanism by which FSP27 regulates these gene expression programs is not clear, our study strongly suggests that FSP27 is an important molecular determinant in controlling gene expression networks and in maintaining white adipocyte identity.

## Methods

### Animal breeding and maintenance

*FSP27-*deficient (*FSP27*^-/-^) mouse breeding and maintenance were essentially performed as described previously [42]. The Leptin/*FSP27 *double deficiency (*ob/ob/FSP27*^-/-^) mice were generated by crossing *FSP27*^-/- ^mice with heterozygous *ob/+ *mice (B6.V-Lepob/J) in a C57BL/6 background that were obtained from The Jackson Laboratories (Bar Harbor, ME). Genotyping for *FSP27 *and *leptin *deficiency in the offspring was performed as described previously [42,63]. The *ob/ob/FSP27*^-/- ^littermates were compared with their leptin deficient (*ob/ob*) littermates. The *FSP27*^-/- ^and *ob/ob/FSP27*^-/- ^mice were fed a normal chow diet (5053, PicoLab Rodent Diet 20). Mouse experiments were carried out in the animal facility in the School of Life Sciences, Tsinghua University. Mouse handling procedures were in accordance with the Responsible Care and Use of Laboratory Animals (RCULA) guidelines set by Tsinghua University. All research and experimental protocols involving mice were reviewed and approved by the animal research committee of Tsinghua University.

### RNA extraction, RT-PCR, semi-quantitative real-time RT-PCR and microarray analyses

Gonadal WAT or interscapular BAT from 3- or 9-month-old mice were dissected and quickly frozen in liquid nitrogen upon sacrifice and then stored at -80°C for subsequent total RNA extraction. Total RNA from gonadal WAT or interscapular BAT was isolated individually using the TRIzol reagent (Invitrogen, USA) and quantified spectrophotometrically at 260/280 nm. The integrity of all RNA samples was evaluated on a 1% agarose gel, and all RNA samples were found to be pure with no degradation caused by the isolation procedure. For semi-quantitative real-time RT-PCR (qPCR) analysis, RNA isolated from four individual animals for each genotype was used for independent qPCR analysis. The first-strand cDNAs were synthesized from 2 μg of total RNA in a total volume of 20 μl using oligo-(dT)_20 _primers and the Superscript III RT kit at 50°C for 60 mins according to the manufacturer's protocol (Invitrogen, USA). The reaction was inactivated by incubating the reaction mixture at 70°C for 15 minutes, followed by the removal of RNA complementary to the cDNA with 1 μl (2 units) of E. coli RNase H (Invitrogen, USA) at 37°C for 20 minutes. The cDNA samples were then diluted with sterile deionized water to a total volume of 200 μl, and 2 μl was used for each qPCR reaction.

qPCR analysis was performed using the ABI SYBR Green PCR Master Mix in the MX3000P real-time PCR system (Stratagene, USA) according to the manufacturer's protocol. Primer sequences, annealing temperatures and the size of each amplified PCR product are given in Additional file 7. A BLAST search against the mouse genome using the electronic PCR program from the NCBI Genome Database of the primer pairs confirmed that no genomic or pseudogene PCR products would be amplified. β-actin was used as an internal control for the qPCR analyses. To validate its reliability during the analyses, another common internal control, cyclophilin (an ER specific protein), was used to evaluate the expression levels of β-actin. Its expression was similar between wild-type and *FSP27-*deficient mice. In addition, the expression levels of representative genes (leptin and Collagen-6α1) using cyclophilin as the internal control were similar to those using β-actin as the internal control, indicating that β-actin was a reliable internal control for the normalization of our qPCR results (Additional file 8).

For microarray analyses, equal amounts of total RNA from five pairs of three-month-old male *FSP27-*deficient mice were combined to form RNA pools (total amount of 45 μg). Affymetrix gene chips (Mouse Genome 430 2.0A arrays, Affymetrix, USA) were used for hybridization and data collection. The protocol was performed by the Microarray Facility at the Institute of Molecular and Cell Biology in Singapore. The microarray data were processed with the Affymetrix GeneChip Operating Software (GCOS) and submitted to the GEO repository for the Series record (GSE22693). The gene set enrichment analysis (GSEA) was performed as previously described [64]. The microarray data from *PPAR*α-deficient WAT, *PGC1α/PGC1β *double-deficient brown fat cells and PPARγ2 over-expressing NIH3T3 cells were extracted from the Gene Expression Omnibus with the accession numbers GSE2131[65], GSE5042 [50] and GSE2192 [66], respectively.

### Western blot analysis

Cell lysates from the WAT of wild type and *FSP27*^-/- ^3-month-old male mice were obtained by homogenizing the tissues in RIPA buffer (20 mM HEPES-KOH pH 7.5, 150 mM NaCl, 1 mM EDTA, 10% Glycerol, 0.5% sodium deoxycholate, 1% NP40, 0.1% SDS and protease inhibitor) and subsequently used for western blot analysis as previously described [36]. Antibodies against β-actin (Sigma, USA) as well as β3-AR and CEBPβ (Santa Cruz, USA) were used for the western blot analyses. Protein bands were visualized by an Enhanced Chemiluminescence detection system and quantified by densitometry analysis using Quantity One (Bio-Rad, USA). The experiments were performed in triplicate.

### Statistical analysis

All data were presented as means ± S.E.M. Differences between groups were assessed by a two-tailed, non-paired or paired Student's t-test using the Graph Pad Prism statistics software (GraphPad Software Inc.)

## Abbreviations

ACC1/2: acetyl-CoA carboxylase 1/2; BAT: brown adipose tissue; β3-AR: beta-3 adrenergic receptor; C2: complement factor 2; CEBPα/β: CCAAT/enhancer binding protein alpha/beta; COL3-α: collagen type 3-alpha; COL6-α1: collagen type 6-alpha1; COX 4: cytochrome oxidase 4; COX 8b: cytochrome C oxidase subunit 8b; CPT1: carnitine palmitoyltransferase I; DIO2: type 2 iodothyronine deiondinase; ECM: extracellular matrix; ELOVL3: elongation of very long chain fatty acid-3; FAS: fatty acid synthase; Fn1: fibronectin 1; FSP27: fat specific protein 27; Gα-s: Gs alpha subunit; HSL: hormone sensitive lipase; LDLR: low density lipoprotein receptor; LPL: lipoprotein lipase; LSDP5: lipid storage droplet protein 5; MEST: mesoderm specific transcript; PGC1α: peroxisome proliferator-activated receptor gamma coactivator 1 alpha; PKACα: protein kinase A catalytic subunit alpha; PPAR: peroxisome proliferator-activated receptor; PRDM16: PR domain containing 16; PREF-1: preadipocyte factor 1; RETNLα: resistin related protein alpha; SMAD4: SMAD family member 4; TAG: triacylglycerol; TCA: tricarboxylic acid cycle; TGF-β: transforming growth factor beta; TGFβ-I: Transforming growth factor beta inducible protein; TGFβ-R2: TGF beta receptor 2; TIMP2/4: tissue inhibitor of metalloprotease 2 and 4; TR3: thyroid hormone receptor; UCP1/2/3: uncoupling protein 1, 2 and 3; WAT: white adipose tissue.

## Authors' contributions

DL maintained the animals, performed mouse genotyping, isolated the mouse tissues, generated the RNA from these tissues and performed the majority of the qPCR experiments. DL was also responsible for the organization of the final manuscript figures. YZ performed the bioinformatic analyses of the microarray data. LX helped maintain the animals, performed western blot analyses, performed data analyses and helped revise the manuscript. YW helped with the qPCR analyses. BX performed some of the qPCR experiments. LZ and LX maintained the *ob/ob/FSP27 *double knock-out mice and provided tissues for the qPCR analyses. ZW helped with the microarray analyses. PL and JS were responsible for the experimental design as well as the data coordination, analysis and interpretation. PL and JS were responsible for the writing, revision and finalization of the manuscript as well as for the decision to submit the manuscript for publication. All authors read and approved the final manuscript.

## Supplementary Material

Additional file 1 **Significantly upregulated pathways in Fsp27-deficient WAT**. Genes are grouped according to their function and listed in the alphabetical order under each heading. The last number represents the total genes in one gene set. The middle is the changed (criteria is designated as fold change between wildtype and Fsp27 -/- mice is more than ± 1.41) gene number, Click here for file

Additional file 2 **Factors that inhibit BAT differentiation were down-regulated in the WAT of FSP27-/- mice**. Click here for file

Additional file 3 **Significantly down-regulated pathways in the WAT of Fsp27-null mice**. Genes are grouped according to their function and listed in alphabetical order under each heading. The last number represents the total number of genes in one gene set. The middle number represents the number of genes with an altered expression pattern (criteria designated as a fold-change between wildtype and Fsp27 -/- mice of more than ± 1.41). The first number represents the number of upregulated or downregulated genes in that gene set. Click here for file

Additional file 4 **Expression of BAT-selective genes and the major regulators in the WAT of young female or old male *FSP27 *deficient mice**. (**A & B**) Relative mRNA levels of BAT-specific genes and the main regulatory factors in the WAT of three-month-old female (**A**) or nine-month-old male **(B) **wild type (+/+) and *FSP27 *null (*FSP2*7^-/-^) mice. Click here for file

Additional file 5 **Comparison of microarray and qPCR analyses**. A total of 52 genes were analyzed by qPCR in the current study and a previous report [Ref [42]]. Among these 52 validated genes, 34 were determined to be up- or down-regulated by microarray analysis, and 30 of those 34 genes (88%) were consistently validated by qPCR. The increased sensitivity of qPCR allowed the detection of alterations in the expression levels of low abundant gene such as PREF-1. Only Smad4 expression showed opposite results in the two analyses. N/A: not available; NC: no change according to the given criteria; +: increase; -: decrease. Click here for file

Additional file 6 **Altered profiles of WAT-selective genes in *FSP27*-deficient WAT**. The list of WAT-selective genes is taken from Reference 30. These genes were determined to be differentially expressed in FSP27-deficient WAT by microarray analysis Click here for file

Additional file 7 **Primer sequences for the genes involved in the qPCR analysis**. Click here for file

Additional file 8 **Validation of β-actin as a reliable internal control for the qPCR data analyses**. (**A**) Relative mRNA levels of leptin, Collagen 6 alpha1 (COL6-α1) and cyclophilin using β-actin as an internal control for the normalization of qPCR results. Reduced levels of leptin and COL6-α1 expression but a similar level of cyclophilin expression were observed in the WAT of *FSP27 *null mice (*FSP27*^-/-^) compared with those of wild type mice (+/+). (**B) **Relative mRNA levels of leptin, COL6-α1 and β-actin using cyclophilin as an internal control for the normalization of qPCR results. Reduced levels of leptin and COL6-α1 expression but a similar level of β-actin expression in the WAT of *FSP27 *null mice (*FSP27*^-/-^) compared with those of wild type mice (+/+). Both data sets are consistent and validate the use of β-actin as a reliable internal control for qPCR analysis. Click here for file
