# Supplementary figures and images for: Regulation of gene expression by FSP27 in white and brown adipose tissue
Source: BMC Genomics. 2010 Jul 22;11:446. doi: 10.1186/1471-2164-11-446 (PMC3091643; doi:10.1186/1471-2164-11-446)

**A**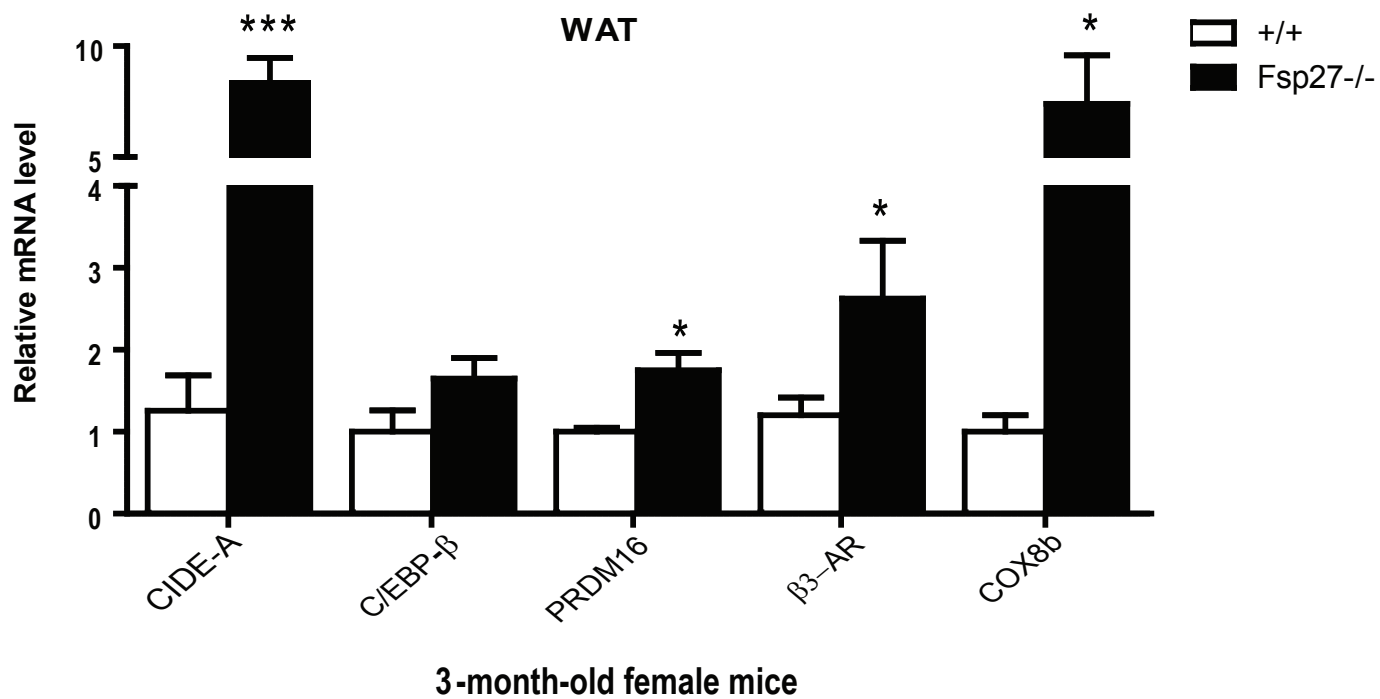**B**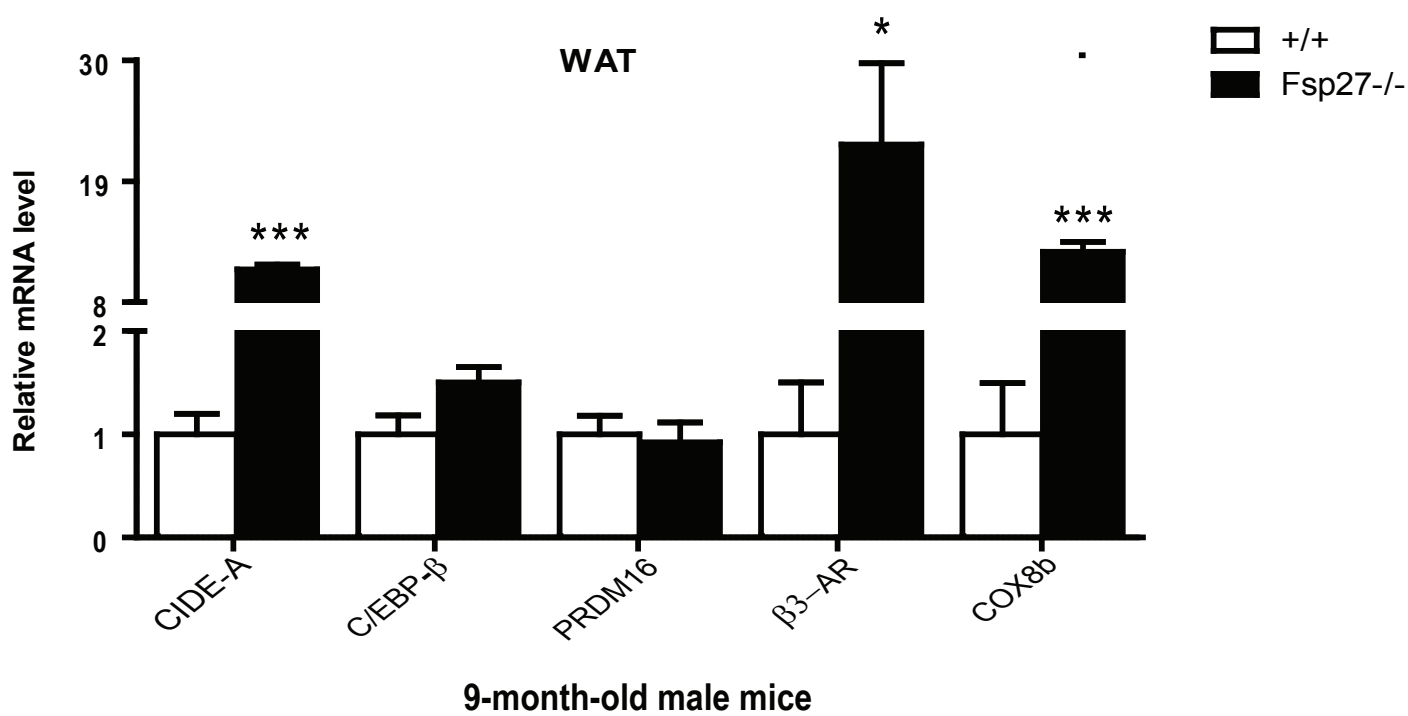

Supplement: Additional file 4 — Expression of BAT-selective genes and the major regulators in the WAT of young female or old male FSP27 deficient mice. (A & B) Relative mRNA levels of BAT-specific genes and the main regulatory factors in the WAT of three-month-old female (A) or nine-month-old male (B) wild type (+/+) and FSP27 null (FSP27-/-) mice. [file 1471-2164-11-446-S4.PDF]

**A**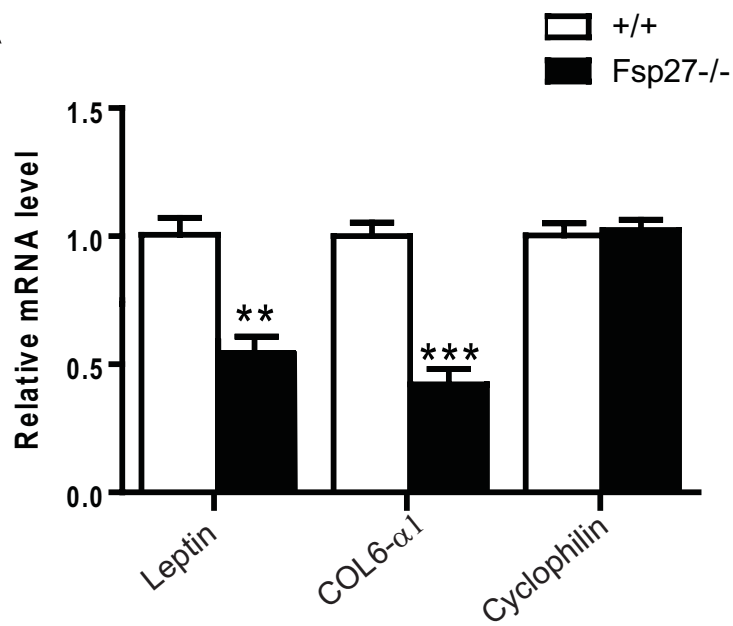**B**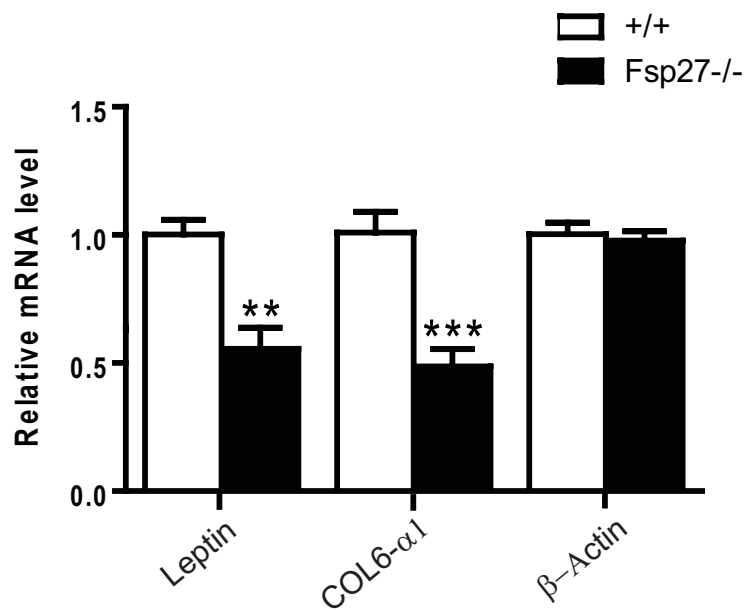

Supplement: Additional file 8 — Validation of β-actin as a reliable internal control for the qPCR data analyses. (A) Relative mRNA levels of leptin, Collagen 6 alpha1 (COL6-α1) and cyclophilin using β-actin as an internal control for the normalization of qPCR results. Reduced levels of leptin and COL6-α1 expression but a similar level of cyclophilin expression were observed in the WAT of FSP27 null mice (FSP27-/-) compared with those of wild type mice (+/+). (B) Relative mRNA levels of leptin, COL6-α1 and β-actin using cyclophilin as an internal control for the normalization of qPCR results. Reduced levels of leptin and COL6-α1 expression but a similar level of β-actin expression in the WAT of FSP27 null mice (FSP27-/-) compared with those of wild type mice (+/+). Both data sets are consistent and validate the use of β-actin as a reliable internal control for qPCR analysis. [file 1471-2164-11-446-S8.PDF]
