# Supplementary material for: Regulation of gene expression by FSP27 in white and brown adipose tissue
Source: BMC Genomics. 2010 Jul 22;11:446. doi: 10.1186/1471-2164-11-446 (PMC3091643; doi:10.1186/1471-2164-11-446)
Supplement: Additional file 7 — Primer sequences for the genes involved in the qPCR analysis. [file 1471-2164-11-446-S7.DOC]

| Gene names | Gene Full name | Forward Primer (5’to 3’) | Reverse Primer (5’to 3’) | PCR Size  (bp) | Annealing Tempreture  (°C) |
| --- | --- | --- | --- | --- | --- |
| ACC1 | [acety1-CoA carboxylase 1](javascript:if(window.name=='') { window.location.href='./nil'; } else { doaction(null, 3121593, 139); }) | AGCTGATCCTGCGAACCT | GCCAAGCGGATGTAAACT | 432 | 62 |
| ACC2 | [acety1-CoA carboxylase 1](javascript:if(window.name=='') { window.location.href='./nil'; } else { doaction(null, 3121593, 139); }) | GTGTTGGACTCTCAAGGACAG | GGATGATGTGGGAGTTTTCCA | 630 | 62 |
| adipsin | adipsin | TGCATGGATGGAGTGACGGATGA | GGTTCCACTTCTTTGTCCTCGTAT | 129 | 62 |
| adiponectin | adiponectin | GGCAGGAAAGGAGAACCTGG | GCCTTGTCCTTCTTGAAGAG | 248 | 62 |
| C2 | complement component 2 | CGGTGGTAATTTCACCCTCAG | GGTGTGATGTGAGCTAGACCT | 148 | 62 |
| CEBP-α | CCAAT/enhancer binding protein alpha | CCGGGAGAACTCTAACTC | GATGTAGGCGCTGATGT | 225 | 62 |
| CEBP-β | CCAAT/enhancer binding protein bete | GCGCGAGCGCAACAACATC | TGCTTGAACAAGTTCCGCAG | 168 | 62 |
| cidea | cell death-inducing DNA fragmentation factor, alpha subunit-like effector A | TGACATTCATGGGATTGCAGAC | GGCCAGTTGTGATGACTAAGAC | 170 | 62 |
| CPT1 | carnitine palmitoyltransferase I | ACCACTGGCCGCATGT | CTCCATGGCGTAGTAGTTGCT | 478 | 62 |
| COL3α1 | collagen, type III, alpha 1 | ACGTAGATGAATTGGGATGCAG | GGGTTGGGGCAGTCTAGTG | 154 | 62 |
| COL6α1 | collagen, type VI, alpha 1 | GATGAGGGTGAAGTGGGAGA | CAGCACGAAGAGGATGTCAA | 219 | 62 |
| COXIV | cytochrome oxidase 4 | CGGCGTGACTACCCCTTG | TGAGGGATGGGGCCATACA | 253 | 62 |
| COX8b | [cytochrome c oxidase subunit VIII](javascript:if(window.name=='') {{ window.location.href='./nil'; }} else {{ open_HOME('/UniPub/iHOP/go?ID1=122905'); }} ) | TGTGGGGATCTCAGCCATAGT | AGTGGGCTAAGACCCATCCTG | 62 | 62 |
| cyclophilin | 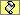 peptidylprolyl isomerase B | GGCTCCGTCGTCTTCCTTTT | ACTCGTCCTACAGATTCATCTCC | 122 | 62 |
| Cyto-C | cytochrome c, somatic | CCAAATCTCCACGGTCTGTTC | ATCAGGGTATCCTCTCCCCAG | 107 | 62 |
| ELOVL3 | elongation of very long chain fatty acids-like 3 | TTCTCACGCGGGTTAAAAATGG | GAGCAACAGATAGACGACCAC | 126 | 62 |
| FAS | fatty acid synthase | TCCAAGACTGACTCGGCTACTGAC | GCAGCCAGGTTCGGAATGCTATC | 261 | 62 |
| FN1 | Fibronectin | CTCATCAGCATCCAGCAG | GGGAGCAGGTCAGGAATG | 302 | 60 |
| Gs-α | G protein α subunit | ACTAAAGTGCAGGACATCAAA | GTCTCAAAGATTCCAGAGGTC | 360 | 62 |
| HSL | hormone sensitive lipase | CCTCATGGCTCAACTCC | GGTTCTTGACTATGGGTGA | 435 | 62 |
| Leptin | leptin | GAGACCCCTGTGTCGGTTC | CTGCGTGTGTGAAATGTCATTG | 139 | 62 |
| LSDP5 | [lipid storage droplet protein 5](javascript:if(window.name=='') { window.location.href='./nil'; } else { doaction(null, 3121593, 139); }) | TGTCCAGTGCTTACAACTCGG | CAGGGCACAGGTAGTCACAC | 101 | 60 |
| LDLR | low density lipoprotein receptor | AGGAGCAGCCACATGGTATGA | CTGTCTTGAGGGGTAGGTAT | 210 | 62 |
| LPL | lipoprotein lipase | GCCCTACAAAGTGTTCCATTAACC | TCATGAGCAGTTCTCCGATGTCCA | 205 | 62 |
| MEST | mesoderm specific transcript | GTGGTGGGTCCAAGTAGGG | AAGCACAACTATCTCAGGGCT | 214 | 60 |
| Perilipin | Perilipin | GGCCAACACTCTTTCTCGACACA | ACTCTTCTTCCTCCTCCTCGTCGT | 245 | 62 |
| PREF1 | Preadipocyte factor 1 | GCGGCTATGGGCTCACCTACC | TCCTCGCCGCTGTTATACTGC | 294 | 62 |
| PRDM16 | PR domain containing 16 | CAGCACGGTGAAGCCATTC | GCGTGCATCCGCTTGTG | 86 | 62 |
| PKAC-α | cAMP dependent protein kinase, catalytic subunit α | ATCGTCCTGACCTTTGAGTAT | ACTTGTGGTTCTTGATGTCAT | 440 | 62 |
| RETNLα | resistin like alpha | TCCCAGTGAATACTGATGAGA | CCACTCTGGATCTCCCAAGA | 214 | 60 |
| SMAD4 | MAD homolog 4 (Drosophila) | ACACCAACAAGTAACGATGCC | GCAAAGGTTTCACTTTCCCCA | 83 | 62 |
| TGFβR2 | transforming growth factor, beta receptor II | ATGGAAGAGTGCAACGATTACAT | TGGCGCAGTTGTCACTGAAAT | 235 | 62 |
| TGFβi | transforming growth factor, beta induced | CAGCACGGCCCCAATGTAT | GGGACCTTTTCATATCCAGGACA | 149 | 62 |
| TGFβ1 | [transforming growth factor, beta 1](javascript:if(window.name=='') { window.location.href='./nil'; } else { doaction(null, 3121593, 139); }) | TGACGTCACTGGAGTTGTACGG | GGTTCATGTCATGGATGGTGC | 170 | 62 |
| TNFα | tumor necrosis factor | CCAGACCCTCACTAGATCA | CACTTGGTGGTTTGCTACGAC | 77 | 62 |
| TIMP2 | tissue inhibitor of metalloproteinase 2 | TCAGAGCCAAAGCAGTGAGC | GCCGTGTAGATAAACTCGATGTC | 142 | 60 |
| TIMP4 | tissue inhibitor of metalloproteinase 4 | TGTGGCTGCCAAATCACCA | TCATGCAGACATAGTGCTGGG | 130 | 62 |
| TR3 | nuclear receptor subfamily 4, group A, member 1 | TTGAGTTCGGCAAGCCTACC | GTGTACCCGTCCATGAAGGTG | 100 | 62 |
| UCP1 | uncoupling protein 1 | AGATCTTCTCAGCCGGAGTTT | CTGTACAGTTTCGGCAATCCT | 180 | 62 |
| UCP2 | uncoupling protein 2 | GTGACTTGCCCAGGAATCTGA | ACTTGGGAGGCAGATGCAGGTAGA | 215 | 62 |
| UCP3 | uncoupling protein 3 | GATCAGCCCACAGTGTTGTC | CTGTTATCCCAATCACGCTC | 241 | 62 |
| β3-AR | β3-Adrenoceptor | CGAGACTACAGACCATAACCA | ATAGACGAAGAGCATCACAAG | 475 | 62 |
